# Characterization and expression profiling of glutathione *S*-transferases in the diamondback moth, *Plutella xylostella* (L.)

**DOI:** 10.1186/s12864-015-1343-5

**Published:** 2015-03-05

**Authors:** Yanchun You, Miao Xie, Nana Ren, Xuemin Cheng, Jianyu Li, Xiaoli Ma, Minming Zou, Liette Vasseur, Geoff M Gurr, Minsheng You

**Affiliations:** Institute of Applied Ecology and Research Centre for Biodiversity and Eco-Safety, Fujian Agriculture and Forestry University, Fuzhou, 350002 China; College of Life Science, Fujian Agriculture and Forestry University, Fuzhou, 350002 China; Key Laboratory of Integrated Pest Management of Fujian and Taiwan, China Ministry of Agriculture, Fuzhou, 350002 China; Department of Biological Sciences, Brock University, 500 Glenridge Avenue, St. Catharines, ON L2S 3A1 Canada; EH Graham Centre, Charles Sturt University, Orange, NSW 2800 Australia

**Keywords:** Transcriptome analysis, qRT-PCR, Phylogenetic analysis, Insect pest, Lepidoptera

## Abstract

**Background:**

Glutathione *S*-transferases (GSTs) are multifunctional detoxification enzymes that play important roles in insects. The completion of several insect genome projects has enabled the identification and characterization of GST genes over recent years. This study presents a genome-wide investigation of the diamondback moth (DBM), *Plutella xylostella*, a species in which the GSTs are of special importance because this pest is highly resistant to many insecticides.

**Results:**

A total of 22 putative cytosolic GSTs were identified from a published *P. xylostella* genome and grouped into 6 subclasses (with two unclassified). Delta, Epsilon and Omega GSTs were numerically superior with 5 genes for each of the subclasses. The resulting phylogenetic tree showed that the *P. xylostella* GSTs were all clustered into Lepidoptera-specific branches. Intron sites and phases as well as GSH binding sites were strongly conserved within each of the subclasses in the GSTs of *P. xylostella*. Transcriptome-, RNA-seq- and qRT-PCR-based analyses showed that the GST genes were developmental stage- and strain-specifically expressed. Most of the highly expressed genes in insecticide resistant strains were also predominantly expressed in the Malpighian tubules, midgut or epidermis.

**Conclusions:**

To date, this is the most comprehensive study on genome-wide identification, characterization and expression profiling of the GST family in *P. xylostella*. The diversified features and expression patterns of the GSTs are inferred to be associated with the capacity of this species to develop resistance to a wide range of pesticides and biological toxins. Our findings provide a base for functional research on specific GST genes, a better understanding of the evolution of insecticide resistance, and strategies for more sustainable management of the pest.

**Electronic supplementary material:**

The online version of this article (doi:10.1186/s12864-015-1343-5) contains supplementary material, which is available to authorized users.

## Background

The diamondback moth (DBM), *Plutella xylostella* (L.) (Lepidoptera: Plutellidae), is a world-wide destructive pest of wild and cultivated crucifers [1]. The larvae feed on cruciferous plants and may cause significant reductions in yield and quality of economically important crops such as canola and cabbage. Historical reliance on insecticides has led to the rapid development of resistance in *P. xylostella* populations [2], making it difficult to control.

Several studies have examined the potential mechanisms underlying the development of insecticide resistance in *P. xylostella* [3-5]. One of the proposed mechanisms is metabolic resistance through the multifunctional glutathione *S*-transferases (GSTs, EC2.5.1.18). These enzymes can catalyze electrophilic compounds, making them water soluble and readily excreted [6]. GSTs are known more generally by insects to detoxify various xenobiotics, including insecticides and plant allelochemicals [7]. The recent work has focused on the potential role of GSTs in oxidative stress responses [6,8-11].

Insect GSTs are classified as cytosolic and microsomal. The number of microsomal GSTs is much lower than that of cytosolic GSTs, which have been grouped into six subclasses [12]. Delta and Epsilon subclasses are insect specific, while the other four subclasses, Omega, Sigma, Theta, and Zeta, are found in various animal taxa [10,13,14].

GSTs are involved in the resistance of insects to organophosphate (OPs), chlorine, and pyrethroid insecticides [15,16]. Recombinant GST enzymes from *P. xylostella* and *Drosophila melanogaster* have been shown to play a role in the metabolism of organophosphate insecticides [17,18]. It has been suggested that, under elevated GST activity conditions, *Anopheles subpictus* can detoxify fenitrooxon activation products, leading to organophosphate resistance [19]. The silkworm Zeta GST recombinant protein (rbmGSTz) has been found to initiate the dechlorination of permethrin and to be abundantly distributed in a permethrin-resistant strain [20]. Similarly, an Omega GST is highly expressed in a fenitrothion-resistant strain of silkworm and its recombinant protein (rbmGSTo) shows high affinity with organophosphate insecticides, indicating that it may contribute to insecticide resistance and oxidative stress responses [21]. The antennae-specific GST was found being involved with detoxification of xenobiotics and detection of sex pheromones in *Manduca sexta* [22].

The GSTs were found to be one of the major enzyme families in the *P. xylostella* genome and to be linked to detoxification of plant defense compounds and insecticides [23]. A recent study on the identification and characterization of multiple glutathione *S*-transferase genes [24] based on the DBM transcriptome database [24,25] provides a primary base for further investigation of this important gene family. In the present study, the *P. xylostella* GSTs (PxGSTs) were identified and compared with the equivalent information from published insect genomes to better reveal their phylogenetic relationships and intron-exon organization. We profiled and analyzed expression patterns of the PxGSTs using the published transcriptome [26] and reverse transcription-quantitative polymerase chain reaction (qRT-PCR) in different life stages and tissues from insecticide susceptible or resistant strains. We then examined the major characteristics of GST subclasses and some particular GST genes in relation to their potential roles in *P. xylostella* insecticide resistance.

## Results and discussion

### Identification of the PxGSTs

Queries for PxGSTs were done against the amino acid sequences from the other insects: *Drosophila melanogaster* (*Dm*), *Culex quinquefasciatus* (*Cq*)*, Aedes aegypti* (*Aa*)*, Anopheles gambiae* (*Ag*) (Diptera)*, Tribolium castaneum* (*Tc*) (Coleoptera)*, Apis mellifera (Am), Nasonia vitripennis* (*Nv*) (Hymenoptea), *Pediculus humanus* (*Ph*)*, Acyrthosiphon pisum* (*Ap*) (Exopterogota)*,* and *Bombyx mori* (*Bm*) (Lepidoptera). Twenty-two putative cytosolic GST genes with full-length sequence were identified from our *P. xylostella* (*Px*) genome [23,27] (Table 1) and further validated by cloning and sequencing. Using the listed gene IDs in Table 1, the coding sequences (Additional file 1), inferred amino acid sequences (Additional file 2) and genomic DNA sequences (Additional file 3) can be found in the published DBM genomic database (DBM-DB: http://iae.fafu.edu.cn/DBM/family/PxGSTs.php) [27]. Compared to the previous DBM GSTs [24] identified from a published *P. xylostella* transcriptomic database, we identified three additional genes. The PxGSTs represented all six subclasses found in other insects [28-30], plus two genes that could not be assigned to any one of the known subclasses, labeled as unclassified. Numbers of GSTs varied greatly across insect species. GSTs were expanded in the Diptera and Coleoptera, with a relatively larger number of total genes than that in the species of Lepidoptera, Hymenoptera and Exopterogota (Table 2). The number of GSTs in *P. xylostella* was close to that of another lepidopteran species, *B. mori*. The two insect-specific GST subclasses (Delta and Epsilon) were numerically superior, accounting for > 50% of the entire cytosolic GSTs in Diptera and Coleoptera and ~ 50% in Lepidoptera (Table 2). This indicates that the GSTs in the Delta and Epsilon subclasses have a greater general trend of duplication than the GSTs in the other four subclasses as previously reported by Friedman [30].

**Table 1:** **Description of 22 identified cytosolic GSTs in the** ***P. xylostella*** **genome**

**Gene name**	**ORF (bp)**	**Protein (AA)**	**Gene size (bp)**	**Scaffold/orientation**	**Gene ID** ^**a**^
*PxGSTd1* ^b^	654	217	2534	38/+	Px010343
*PxGSTd*2	648	215	3031	75/+	Px015896
*PxGSTd*3^b^	660	219	1640	75/-	Px015897
*PxGSTd*4	672	223	2321	221/+	Px006286
*PxGSTd5*	672	223	2294	73/-	Px015631
*PxGSTe1*	699	232	1823	66/+	Px014816
*PxGSTe2* ^b^	684	227	3497	363/-	Px010078
*PxGSTe3*	687	228	1699	41/+	Px011036
*PxGSTe4*	663	220	2922	216/+	Px006106
*PxGSTe5*	651	216	4863	216/+	Px006105
*PxGSTo1*	768	255	2649	85/+	Px016897
*PxGSTo2* ^b^	750	249	6717	554/+	Px016898
*PxGSTo3*	726	241	3721	25/+	Px007118
*PxGSTo4*	750	249	750	7/-	Px015266
*PxGSTo5*	738	245	2200	554/+	Px013473
*PxGSTs1*	615	204	2669	320/-	Px009113
*PxGSTs2*	615	204	8124	328/-	Px009257
*PxGSTt1*	654	217	3261	547/-	Px000759
*PxGSTu1* ^b^	693	230	2771	1088/-	Px000790
*PxGSTu2*	648	215	963	408/-	Px010993
*PxGSTz1*	645	214	2450	16/-	Px003659
*PxGSTz2*	642	213	15681	115/+	Px001225

^a^The gene IDs were obtained directly from the published DBM genomic database (DBM-DB: http://iae.fafu.edu.cn/DBM/family/PxGSTs.php). All the coding sequences (CDS) of the PxGST genes have been experimentally validated.

^b^Coding sequences of such genes were incomplete from the DBM-DB, and have been experimentally completed by PCR, as explained in the methodology.

*Px*: *Plutella xylostella*.

**Table 2:** Comparison of GST gene numbers of various insect species*

**Insect species**	**Delta**	**Epsilon**	**Omega**	**Sigma**	**Theta**	**Zeta**	**Unclassified**	**Total**
*P. xylostella*	5	5	5	2	1	2	2	22
*B. mori*	4	8	4	2	1	2	2	23
*C. quinquefasciatus*	17	10	1	2	6	0	3	39
*D. melanogaster*	11	14	5	1	4	2	0	37
*A. gambiae*	12	8	1	1	2	1	3	28
*A. aegypti*	8	8	1	1	4	1	3	27
*T. castaneum*	3	19	3	7	1	1	2	36
*N. vitripennis*	5	0	2	8	3	1	0	19
*A. mellifera*	1	0	1	4	1	1	0	8
*A. pisum*	9	0	2	5	2	0	6	24
*P. humanus*	4	0	1	4	1	1	0	11

*Data were from cited literature: Friedman (2011) [30], Oakeshott *et al*. (2010) [31], Yu *et al*. (2008) [28], Ding *et al*.(2003) [29] and Nair *et al*.(2011) [32].

### Phylogenetic analysis of the PxGSTs

**Figure 1:**
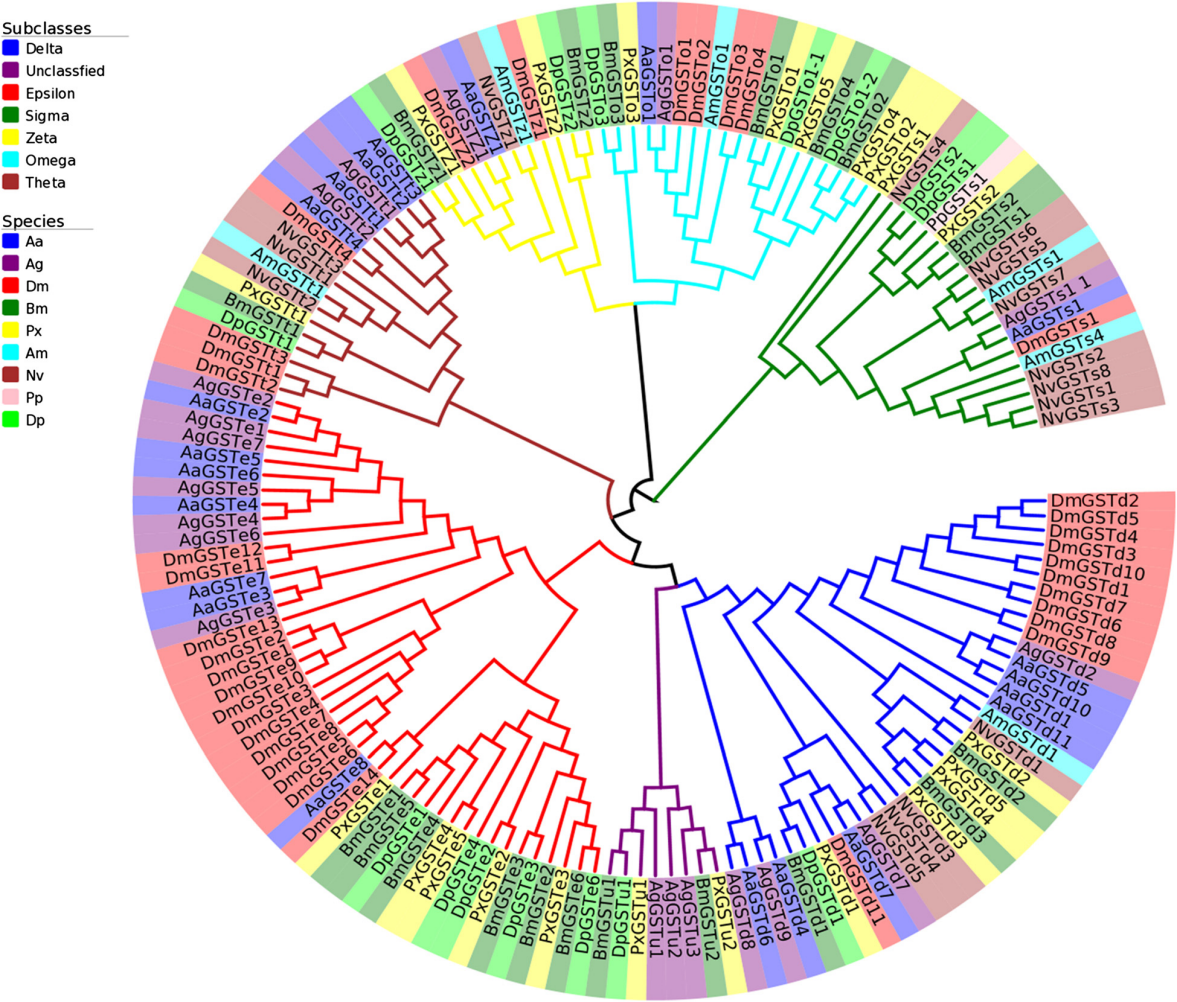
**Unrooted phylogenetic tree of the cytosolic GSTs in nine targeted insect species.** The tree was constructed using neighbor-joining approach with MEGA 5.10 [35] on the basis of Poisson correction amino acid model and pairwise deletion of gaps. Species acronym (*Aa*: *Aedes aegypti*; *Ag*: *Anopheles gambiae*; *Dm*: *Drosophila melanogaster* (Diptera); *Bm*: *Bombyx mori*; *Pp*: *Papilio polyte*s; *Dp*: *Danaus plexippu*s; *Px*: *Plutella xylostella* (Lepidoptera); *Am*: *Apis mellifera*; *Nv*: *Nasonia vitripennis* (Hymenoptea)) was used right before each of the GST genes.

The phylogenetic tree illustrated that the seven subclasses were well clustered into their relevant phylogenetic branches (Figure 1). The unclassified subclass diverged from the Delta subclass, suggesting that they may have similar functions. In all the subclasses, the *P. xylostella* GSTs were all clustered into the Lepidoptera-specific branches. Within a specific subclass, the same genes in different species were first clustered into an upper branch within the phylogenetic tree, suggesting that specific GSTs in different species might have same or similar functions [33,34].

Delta and Epsilon GST subclasses are unique to insects and have been suggested to be implicated in insecticide resistance [10,36,37]. The earlier diverging insects Hymenoptera and Exopterygota do not have Epsilon subclass GSTs. Hymenoptera has a few genes present in the Delta subclass but none for Exopterogota suggesting that these orders may be from an evolutionary older lineage [30]. Our tree suggests that Delta and Epsilon GSTs have diverged more recently from the other subclasses.

The range of amino acid identities in the insect-specific GSTs of *P. xylostella* are fairly variable, ranged from 38.39 ~ 84.75% in Delta and 23.05 ~ 60.91% in Epsilon (Additional file 4: Table S1). Except for *PxGSTe1*, the remaining Epsilon PxGSTs were clustered in a monophyletic clade of Lepidoptera (Figure 1), suggesting a lineage-specific expansion within the Epsilon subclass in lepidopteran order*.*

### Characterization of the PxGST introns

**Figure 2:**
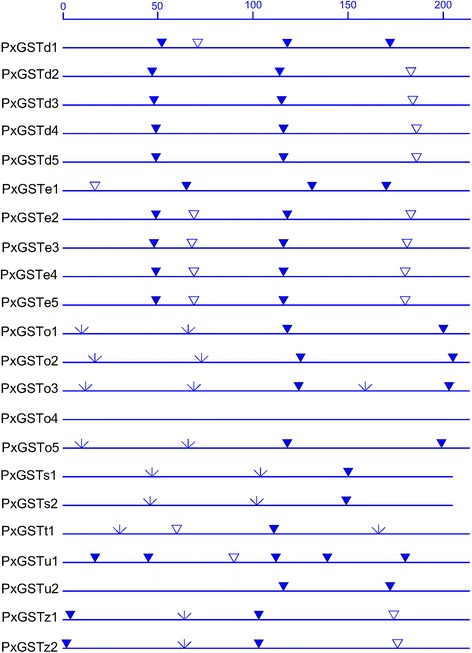
**Location of introns of the PxGST genes.** Phase 0, 1 and 2 introns are shown by inverted filled triangle, arrow and inverted blank triangle, respectively. Phase 0 for a splice site lying between two codons, phase 1 for a splice site lying one base inside a codon in the 3’ direction, and phase 2 for a splice site lying two bases inside the codon in the 3’ direction.

A total of 80 introns were identified in the PxGSTs. Except for one intronless gene (*PxGSTo4*), the intron numbers of individual PxGSTs ranged from 2 to 6 (Figure 2) with an average of 3.6. These numbers are similar to those of *B. mori* GSTs with an average of 3.4 [28] and larger than those of Dipteran (*A. gambiae*) and Coleopteran (*T. castaneum*) GSTs with averages of 1.5 and 2.3, respectively [29,38]. The number of GSTs introns has been shown to vary across insect species. It is thought to be associated with the ability to respond to xenobiotics and endogenous compounds [39].

In the PxGSTs, the splice sites of introns were classified into three phases: 0 with 45 introns, 1 with 17 introns, and 2 with 18 introns, according to their positions in the codons. The phase-1 introns were present only in the Omega, Zeta and Sigma subclasses. Most phase-2 introns were found in insect-specific subclasses (Delta and Epsilon) as well as in Zeta subclass. Most of the PxGST introns spliced in a given site tended to be from the same phase, suggesting that they might be relatively conserved (Figure 2).

Intron sites are similar across different PxGST subclasses. There are three highly conserved sites of introns within the Delta and Epsilon GST subclasses, except for the *PxGSTd1* and *PxGSTe1*. These were between the 47^th^ and 51^st^, the 114^th^ and 118^th^ and the 180^th^ and 186^th^ amino acids. Most of the PxGSTs tended to have a nearby conserved site of the introns located between the 111^th^ and 125^th^ amino acids belonging to phase 0. Both of the intron sites and phases were strongly conserved within Sigma and Zeta subclasses (Figure 2), implying that these genes might have similar functions. There appeared to be a correlation between the intron conservation and the phylogenetic cluster within a given PxGST subclass, indicating that gene structure evolution might be involved in the phylogenetic development of a specific subclass.

Despite the conserved nature of intron sites and phases, the lengths were highly variable in the PxGSTs ranging from 28 to 17,644 bp with a larger proportion ranging from 300 to 399 bp (Additional file 5: Figure S1) and an average of 918 bp. The shortest intron was *PxGSTu1* (28 bp), while the longest were *PxGSTo3* (17,644 bp) and *PxGSTz2* (13,241 bp). A previous study has shown that long introns were considered to involve more functional elements than short introns and could effectively regulate gene expressions, possibly via the formation of pre-mRNA secondary structures [40]. However, the function of the longest introns in *PxGSTo3* and *PxGSTz2* needs to be further investigated.

### GSH and substrate binding sites in the PxGSTs

**Figure 3:**
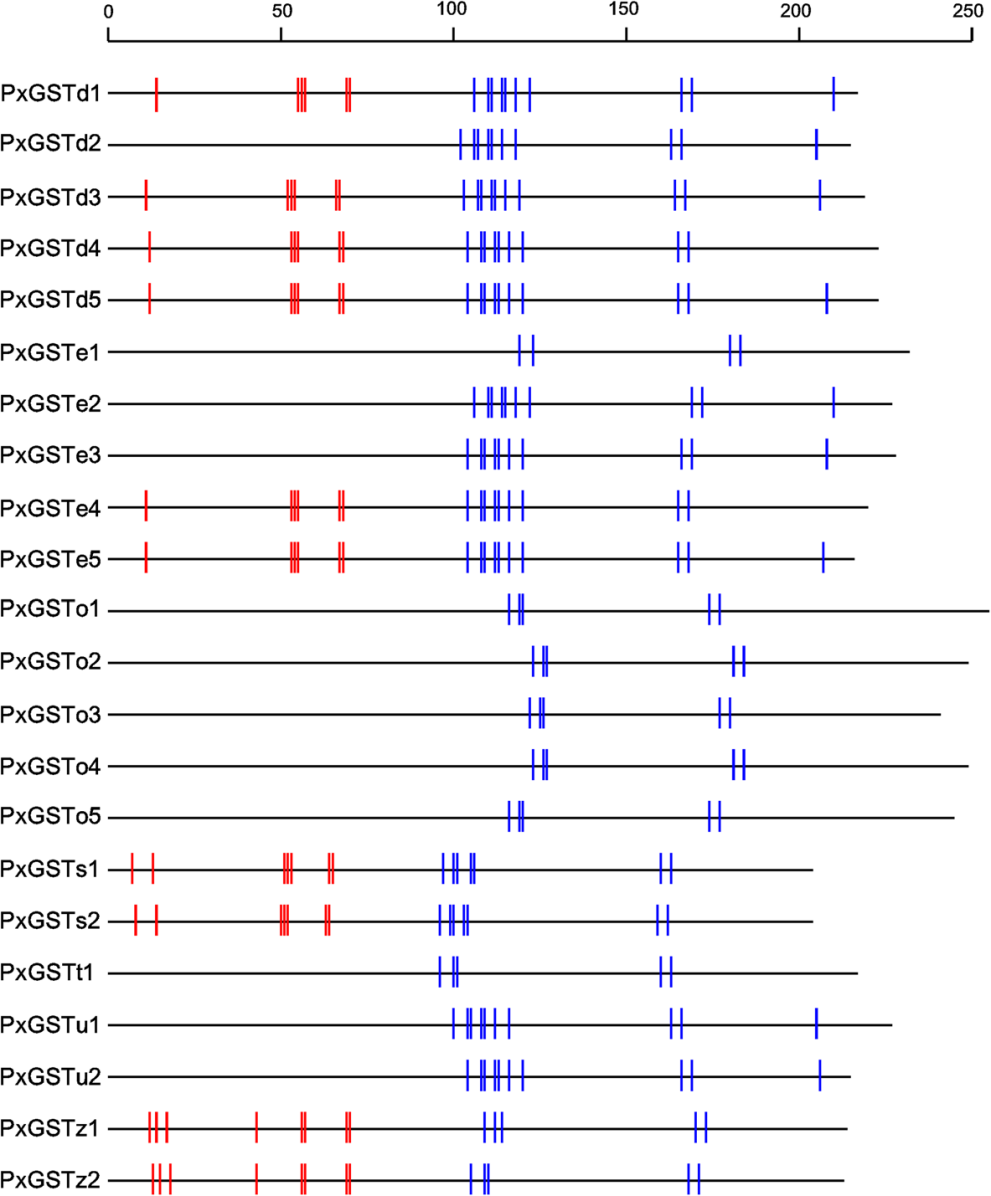
**GSH and substrate binding sites of glutathione** ***S*** **-transferase genes.** The short vertical lines represent functionally conserved residues of GST genes among insect species. Red vertical lines represent the GSH binding sites of GSTs (G sites) and blue vertical lines represent the substrate binding sites GSTs (H sites).

Most of the insect GSTs are composed of a conserved thioredoxin domain containing the GSH binding site (G-site) and a more variable α-helical domain containing the substrate binding site (H-site) [41], and can transfer GSH to a substrate by stabilization of the GSH thiolate [42]. Both G-sites and H-sites among the PxGSTs were analyzed with the NCBI CD-search program, and the results showed that the G-sites appeared fairly conserved while the H-sites were variable among different subclasses (Figure 3, Additional file 6: Table S2). The conserved G-sites indicate their important enzyme functions while the variable H-sites are related to their evolutionary divergence [43]. No G-sites were found for all the genes in Omega GSTs as well as some genes in other subclasses in *P. xylostella* (Figure 3). Such GSTs (without G-site) may act as intracellular ligand transporters as documented in *Nilaparvata lugens* and *Anopheles cracens* [43,44].

## Expression profiling of the PxGSTs

### Stage-specific expression profiling

**Figure 4:**
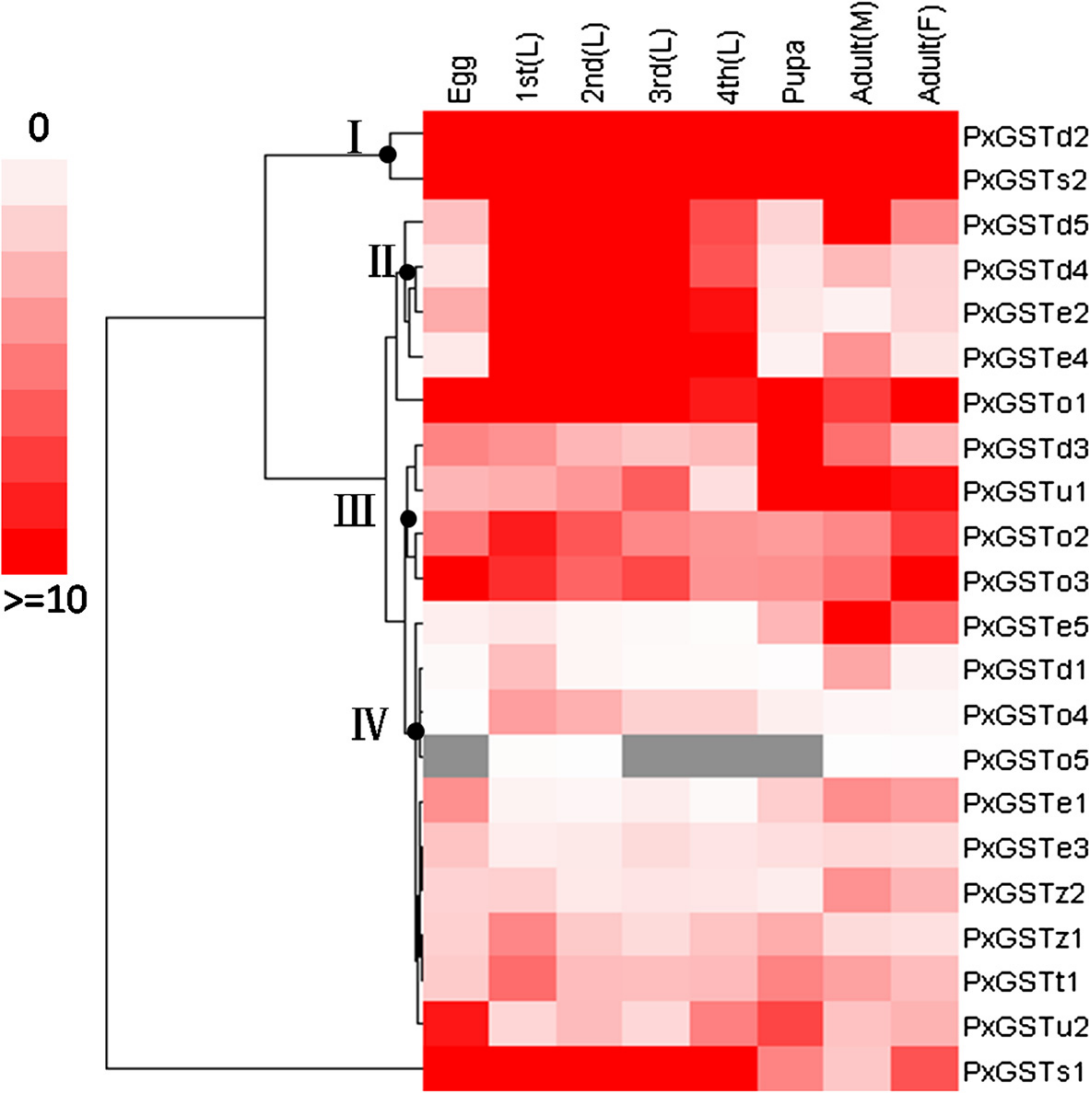
**Expression profiling of the PxGSTs at different developmental stages based on RPKM value.** 1st (L): first instar larva; 2nd (L): second instar larva; 3rd (L): third instar larva; 4th (L): fourth instar larva; F: female; M: male; Gray denoted missing values. The data were obtained from our unpublished RNA-seq data. The RPKM values are presented in Additional file 7: Table S3. The data have been uploaded to the *P. xylostella* genomic database (DBM-DB: http://iae.fafu.edu.cn/DBM/family/PxGSTs.php).

Using our unpublished *P. xylostella* RNA-seq data, expression patterns of the PxGSTs at different developmental stages of the susceptible strain were characterized (Figure 4, Additional file 7: Table S3). The results showed that all 22 PxGSTs could be expressed at different developmental stages, and exhibited gene-differential and stage-specific patterns. Sixteen genes were found to be consistently expressed throughout different stages, two of which (*PxGSTd2* and *PxGSTs2*, Figure 4, I) tended to be expressed with high levels, four (*PxGSTd3*, *PxGSTu1, PxGSTo2*, and *PxGSTo3*) with moderate levels (Figure 4, III) and ten with low levels (Figure 4, IV). Those highly and moderately expressed genes may function as housekeeping genes with potential roles of protecting cells against endogenous oxidative stress or xenobiotics [28]. Four insect-specific PxGSTs (Figure 4, II) were predominantly and highly expressed at in larval (the main feeding stage), indicating that these genes might play important roles in metabolizing plant secondary metabolites [45,46].

**Figure 5:**
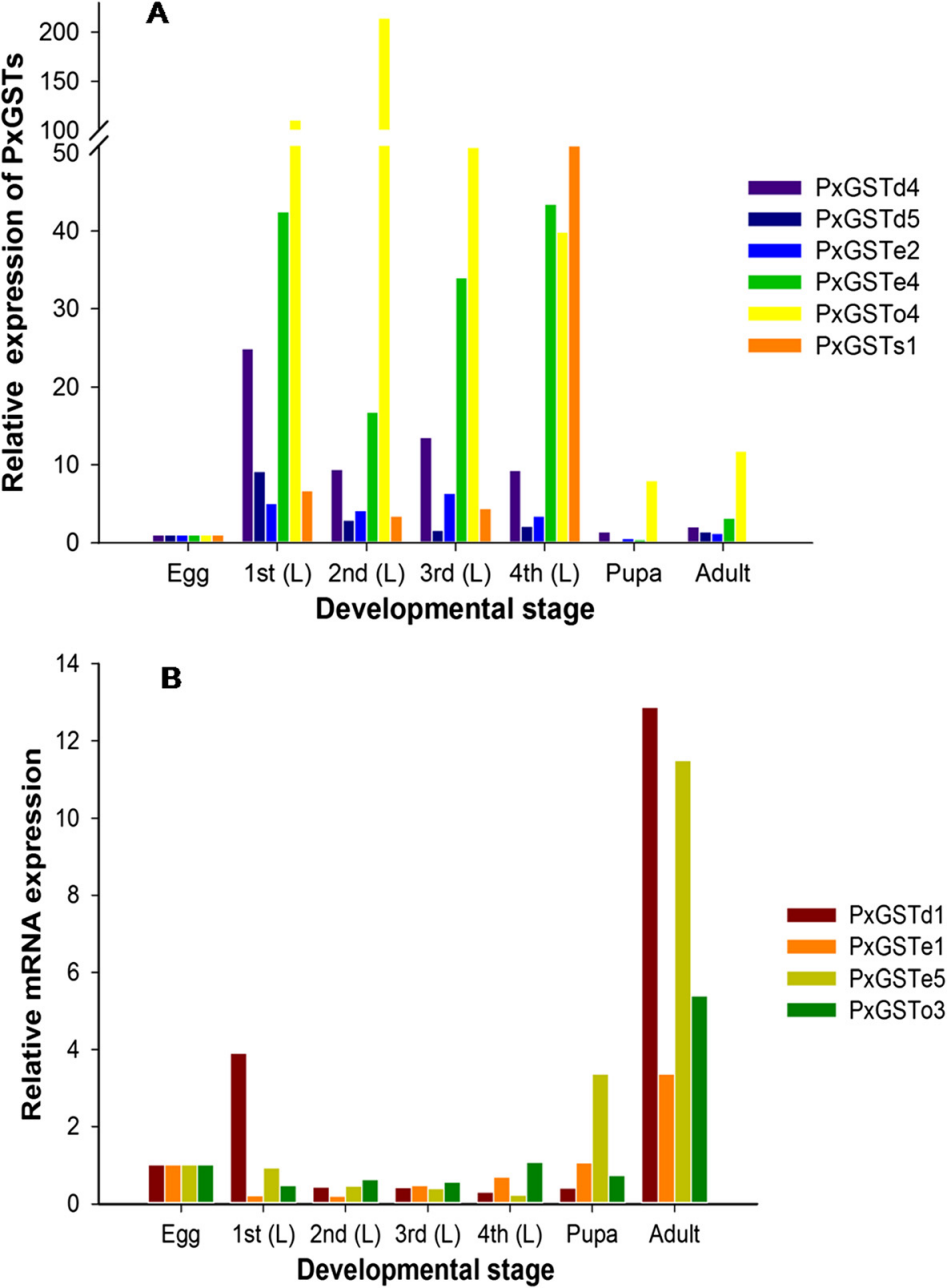
**Expression profiling of selected preferentially expressed PxGSTs at larval (A) and adult (B) stages based on qRT-PCR.** 1st (L): first instar larva; 2nd (L): second instar larva; 3rd (L): third instar larva; 4th (L): fourth instar larva.

Expression profiling with qRT-PCR confirmed that the 22 PxGSTs genes could express at different developmental stages, but exhibited stage-specific patterns. Six genes were predominantly expressed at the larval stage, exhibiting the same patterns based on RPKM value (Figure 4) and qRT-PCR (Figure 5A), which suggests that they might be associated with detoxification of plant defense compounds and insecticides [34,46]. The insect-specific Delta and Epsilon GSTs showed high expression in *P. xylostella* (Figure 5A), while most of these GSTs had little or no expression in the main detoxification organ (fat body) of domesticated *B. mori* with little exposure to insecticides for thousands years [28], suggesting that these genes are associated with the evolution of insecticide resistance as proposed in previous reports [34,45]. *PxGSTd1*, *PxGSTe1*, *PxGSTe5* and *PxGSTo3* were highly expressed in the *P. xylostella* adults (Figure 5B), suggesting that these genes may be involved in odorant processing and/or xenobiotic metabolism [22]. Most of the PxGSTs exhibited low gene expression at the egg stage (Figure 5). Such diversified expression patterns of the PxGSTs imply that GSTs may have multiple functions in *P. xylostella*, as documented in other insects [34,47,48].

### Strain- and tissue-specific expression profiling

**Figure 6:**
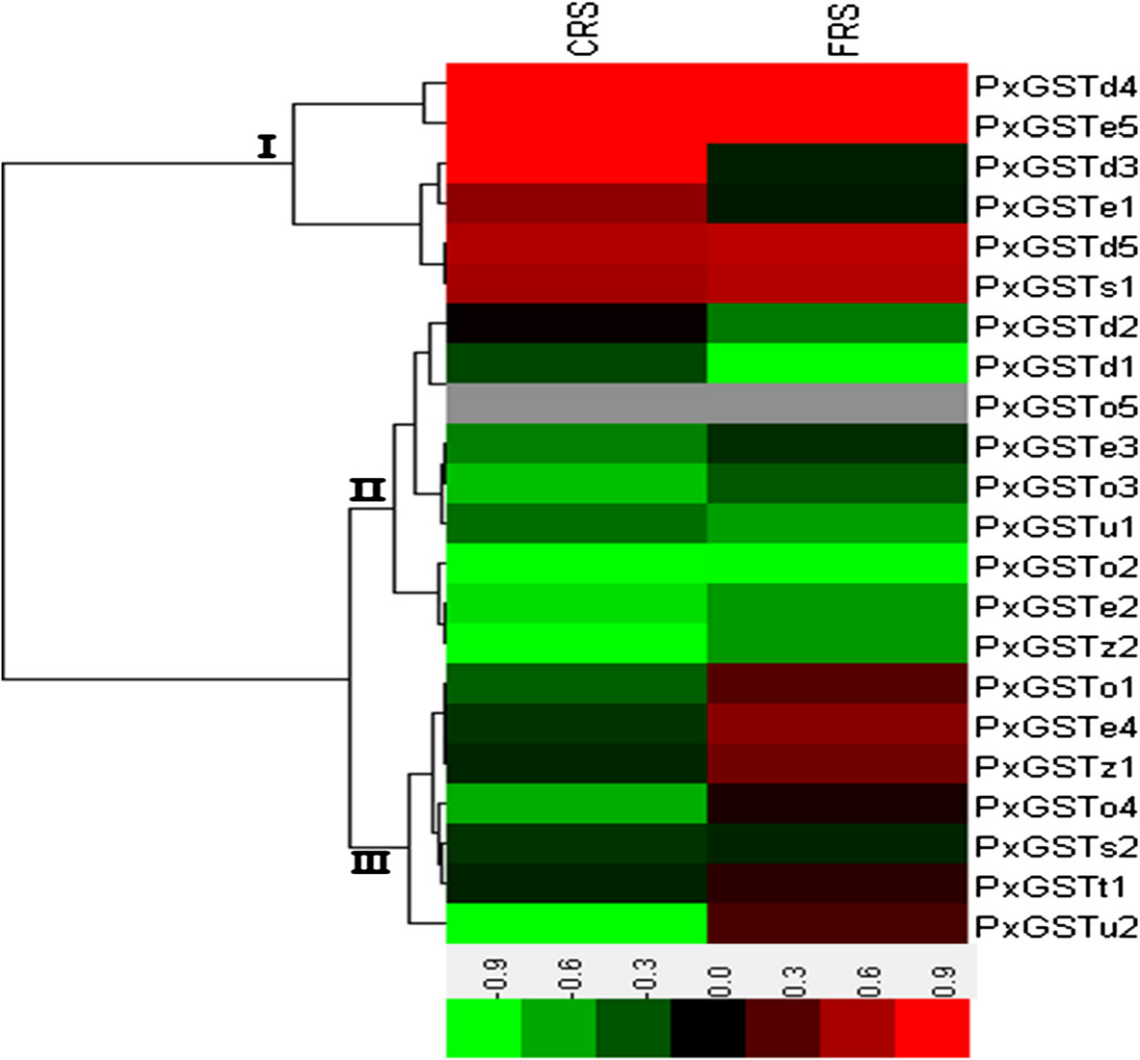
**Differential expressions of the PxGSTs in different resistant strains based on RPKM value.** CRS: chlorpyrifos resistant strain; FRS: fipronil resistant strain. Differential expressions are illustrated by different colors compared to the expression in susceptible strain (SS), with the red representing up-regulated, the green down-regulated and the black no difference with SS. The gray denotes missing values. The data were obtained from our published transcriptome data. The RPKM values are presented in Additional file 8: Table S4. The data have been uploaded to the *P. xylostella* genomic database (DBM-DB: http://iae.fafu.edu.cn/DBM/family/PxGSTs.php).

Based on the DBM transcriptome, all of the PxGSTs were either up- or down-regulated in the insecticide resistant strains when compared to the susceptible strain (SS) (Figure 6, Additional file 8: Table S4). Seven PxGSTs were up-regulated in both of the chlorpyrifos- and fipronil-resistant strains (CRS and FRS) (Figure 6, group I). They are mostly insect specific GSTs (Delta and Epsilon) with the potential function of detoxification. However, seven PxGSTs were down-regulated in CRS and FRS (Figure 6, group II). Most of the rest were up-regulated in FRS, but down- regulated in CRS, possibly reflecting different mechanisms of detoxification between the two strains.

**Figure 7:**
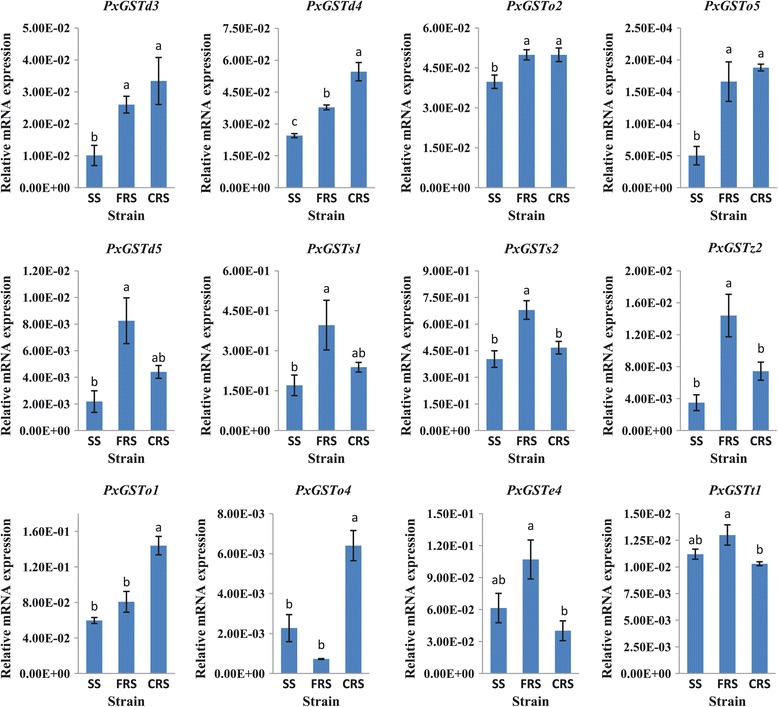
**Expression patterns of PxGSTs in three strains as determined by qRT-PCR.** SS, insecticide susceptible strain; FRS, fipronil resistant strain; CRS, chlorpyrifos resistant strain; Error bars indicate standard errors of the mean. Statistically significant differences were labeled with different letters as evaluated with one-way ANOVA (Duncan’s multiple range test, P < 0.05, n = 3).

The qRT-PCR-based analysis showed that four of the PxGSTs (*PxGSTd3*, *PxGSTd4*, *PxGSTo2* and *PxGSTo5*) showed significantly greater expression in both CRS and FRS. *PxGSTd5*, *PxGSTs1*, *PxGSTs2* and *PxGSTz2* in FRS and *PxGSTo1* and *PxGSTo4* in CRS also had greater gene expressions than in SS. Interestingly, *PxGSTe4* and *PxGSTt1* exhibited higher expressions in FRS but lower expressions in CRS when compared to the SS (Figure 7). Expression of the other 10 PxGSTs were not significantly different among strains. The qRT-PCR-based analysis could not confirm the transcriptome-based expression profiling patterns of all the PxGSTs, which might result from different sampling times when the resistant DBMs (FRS and CRS) were collected for transcriptome sequencing in December 2009, and collected three years later for qRT-PCR. The diversified patterns of strain-specific expressions suggest that the PxGSTs might involve a functionally complex system in response to detoxifying different classes of insecticides [34,49,50].

**Figure 8:**
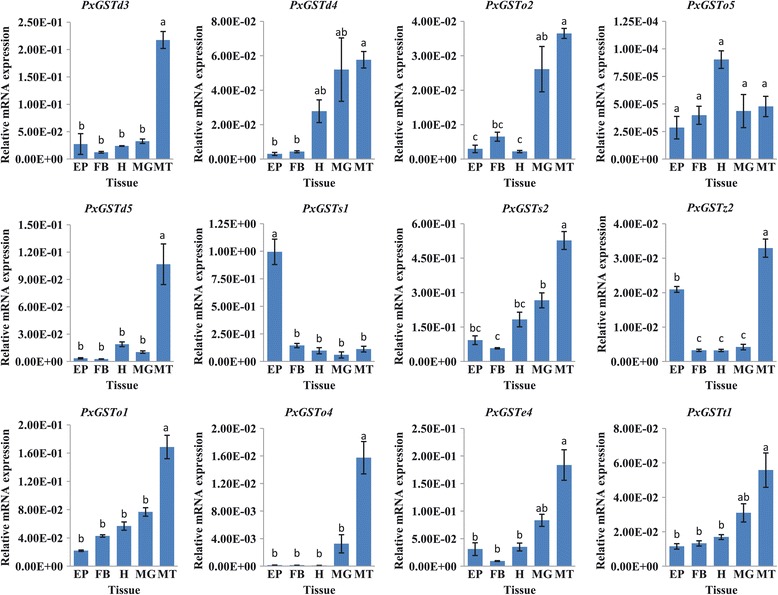
**Expression patterns of PxGSTs in multiple tissues as determined by qRT-PCR.** FRS, fipronil resistant strain; EP, epidermis; FB, fat body; H, head; MG, midgut; MT, Malpighian tubules; Error bars indicate standard errors of the mean. Statistically significant differences were labeled with different letters as evaluated with one-way ANOVA (Duncan’s multiple range test, P < 0.05, n = 3).

Twelve PxGSTs with significantly high expression in resistant strains were further analyzed using qRT-PCR in different tissues of fipronil-resistant strain. The results showed that 6 genes (*PxGSTd3*, *PxGSTd5*, *PxGSTs2*, *PxGSTz2*, *PxGSTo1* and *PxGSTo4*) were significantly more highly expressed in Malpighian tubules, 4 (*PxGSTd4*, *PxGSTo2, PxGSTe4* and *PxGSTt1*) both in Malpighian tubules and midguts and 1 (*PxGSTs1*) in epidermis, except for *PxGSTo5* with no significant different expression in the various tissues (Figure 8). These results further validate the association of the PxGSTs with insecticide detoxification because these tissues were documented to play important roles in digestion and metabolism of xenobiotics in insects [51,52].

## Conclusions

To date, this is the most comprehensive study on genome-wide identification, characterization and expression profile of the GSTs in *P. xylostella*. Twenty-two GSTs were found in *P. xylostella*, which is similar in number to another lepidopteran species, *B. mori*. Variable features and different expression patterns of the genes reveal that the *P. xylostella* GSTs are evolutionary and functionally diversified, and may be involved in the evolution of adaptive capacity in response to environmental variation. Because GST enzymes are considered to be important in insecticide resistance, many of these newly identified genes are potential candidates for inhibiting the pathway of insecticide resistance and targeting lepidopteran-selective insecticides. Thus, further functional research on the PxGSTs is essential to identify the key genes and their roles in xenobiotic detoxification of insects, and understand the mechanisms underlying the insecticide resistance.

## Methods

### Experimental DBM strains

The experimental population of *P. xylostella* was derived from a susceptible strain (SS) that was collected from a vegetable field of Fuzhou (26.08°N, 119.28°E) in 2004 and used for genome sequencing [23]. Since then this initial population was reared on potted radish seedlings *(Raphanus sativus* L.) at 25 ± 1°C, 65 ± 5% RH and L:D = 16:8 h in a separate greenhouse without exposure to insecticides over the past ten years. Two insecticide resistant strains (chlorpyrifos- and fipronil-resistant strains (CRS and FRS)) were selected from this susceptible strain, and detailed in DBM transcriptome [26].

### Identification of *P. xylostella* GST genes

To identify putative GST genes from the DBM genome database [23,27], the GST protein sequences of *D. melanogaster*, *C. quinquefasciatus*, *A. aegypti*, *A. gambiae*, *T. castaneum*, *A. mellifera*, *N. vitripennis*, *A. pisum*, *P. humanus*, *B. mori* and other lepidopteran insects were downloaded from their genome databases [53-57] and/or GenBank (http://www.ncbi.nlm.nih.gov/) and Uniprot (http://www.uniprot.org/). These insect GST protein sequences were used as queries to perform local TBLASTN searches against the DBM genome database. The putative genomic sequences were retrieved, and then predicted using Fgenesh + (http://www.softberry.com/). The DBM GST protein sequences were confirmed using online BLASTP in NCBI.

### Phylogenetic analysis

The GSTs of *A. aegypti* (*Aa*), *A. gambiae* (*Ag*), *D. melanogaster* (*Dm*), *A. mellifera* (*Am*), *N. vitripennis* (*Nv*), *Papilio polytes* (*Pp*), *Danaus plexippus* (*Dp*), *B. mori* (*Bm*) and *P. xylostella* (*Px*) were used for the phylogenetic analysis. Putative amino acid sequences of the GSTs were aligned using Clustal X2.0 [58], and then gaps and missing data were manually trimmed. A phylogenetic tree was constructed with the neighbor-joining method [59] using MEGA 5.10 [35]. Bootstrap analysis with 1,000 replicates was used to evaluate the significance of the nodes. Poisson correction amino acid model and pairwise deletion of gaps were selected for the tree reconstruction.

### RNA extraction and cDNA synthesis

DBM eggs, 1^st^- to 4^th^-instar larvae, pupae and adults from susceptible and resistant strains were frozen in liquid nitrogen. Total RNA was extracted using the RNAiso Plus (Takara, Code: D9108A, Japan). The 4^th^-instar larvae were surface sterilized in 75% ethanol, then dipped in DNAase and RNAse free water and dissected. Tissues (head, midgut, Malpighian tubules, fatbody and epidermis) from resistant strains were briefly immersed in RNAlater™ RNA Stabilization Reagent (QIAGEN, Code: 76104, Germany) then stored at 4°C. Total RNA was extracted with the RNeasy Plus Micro Kit (QIAGEN, Code: 74034, Germany) and RNA concentration was determined using a spectrophotometer (Nanodrop 2000: Thermo, USA).

The cDNA template for PCR was synthesized with 1 μg of total RNA using PrimeScript®RT reagent Kit with gDNA Eraser (Perfect Real Time) (Takara, Code: DRR047A, Japan).

### Validation of gene expression by qRT-PCR

The qRT-PCR primers used in the validation of gene expression were identified based on the encoding sequences of the DBM GSTs (Additional file 9: Table S5). DBM ribosomal protein L8 (RPL8) was used as reference gene for different strains and tissues, and DBM ribosomal protein S4 (RPS4) for different stages/instars. The assays were run in triplicate in CFX96 Touch™ Real-Time PCR Detection Systems (Bio-Rad, USA). PCR amplification was performed in a total reaction volume of 20 μL reaction mixture, containing 20 ng cDNA, 10 μL 2 × SYBR® *Premix Ex Taq*™ (Takara, DRR420A, Japan), 0.2 μM of each primer. PCR was conducted with standard thermal cycle conditions using the two-step qRT-PCR method: an initial denaturation at 95°C for 30s followed by 40 cycles of 3s at 95°C and 30s at 60°C. Specificity of the PCR products was assessed by melting curve analysis for all samples. For each treatment (tissues, strains and developmental stages), there were three biological replicates.

### Statistical analysis

The 2^−ΔCt^ method was used to analyze the qRT-PCR-based expression patterns. One-way ANOVA, using PASW Statistics 18, followed by a Duncan’s multiple range test was used to evaluate significant differences among patterns. The results were presented by mean ± standard deviation of the relative mRNA expressions.

### Availability of supporting data

The nucleic acid sequences and protein sequences have been deposited in the published DBM genomic database (DBM-DB: http://iae.fafu.edu.cn/DBM/family/PxGSTs.php). Other supporting data are presented in Additional file 7: Tables S3 and Additional file 8: Table S4, and also deposited in the same database.

## Additional files

Additional file 1:
**Coding sequences of PxGSTs.**
Additional file 2:
**Amino acid sequences of PxGSTs.**
Additional file 3:
**Genomic DNA sequences of PxGSTs.**
Additional file 4: Table S1. Subclass-based matrix of the amino acid identity among different PxGSTs. Additional file 5: Figure S1. Numerical distribution of intron length in the PxGSTs. Additional file 6: Table S2. GSH and substrate binding sites of the PxGSTs. Additional file 7: Table S3. RPKM of the PxGSTs at different developmental stages obtained from RNA-seq data. Additional file 8: Table S4. RPKM of PxGST genes in different strains obtained from transcriptome data. Additional file 9: Table S5. Primer sequences of qRT-PCR.
