# Supplementary material for: Characterization and expression profiling of glutathione S-transferases in the diamondback moth, Plutella xylostella (L.)
Source: BMC Genomics. 2015 Mar 5;16(1):152. doi: 10.1186/s12864-015-1343-5 (PMC4358871; doi:10.1186/s12864-015-1343-5)
Supplement: Additional file 4: Table S1. — Subclass-based matrix of the amino acid identity among different PxGSTs. [file 12864_2015_1343_MOESM4_ESM.pdf]

Table S1 Subclass-based matrix of the amino acid identity (%) among different PxGSTs

Gene	PxGSTd1	PxGSTd2	PxGSTd3	PxGSTd4	PxGSTd5
PxGSTd1	-	43.89	38.39	44.93	44.05
PxGSTd2		-	63.47	56.50	54.71
PxGSTd3			-	60.09	60.54
PxGSTd4				-	84.75
PxGSTd5					-

Gene	PxGSTe1	PxGSTe2	PxGSTe3	PxGSTe4	PxGSTe4
PxGSTe1	-	23.05	24.08	27.73	29.24
PxGSTe2		-	34.78	40.79	35.53
PxGSTe3			-	37.12	41.56
PxGSTe4				-	60.91
PxGSTe4					-

Gene	PxGSTo1	PxGSTo2	PxGSTo3	PxGSTo4	PxGSTo5
PxGSTo1	-	40.30	26.44	39.16	70.98
PxGSTo2		-	27.56	85.94	37.55
PxGSTo1			-	26.38	27.78
PxGSTo1				-	37.55
PxGSTo1					-

Gene	PxGSTs2	PxGSTz2	PxGSTu2
PxGSTs1	25.96		
PxGSTz1		41.47	
PxGSTu1			29.31
