# Supplementary figures and images for: Characterization and expression profiling of glutathione S-transferases in the diamondback moth, Plutella xylostella (L.)
Source: BMC Genomics. 2015 Mar 5;16(1):152. doi: 10.1186/s12864-015-1343-5 (PMC4358871; doi:10.1186/s12864-015-1343-5)

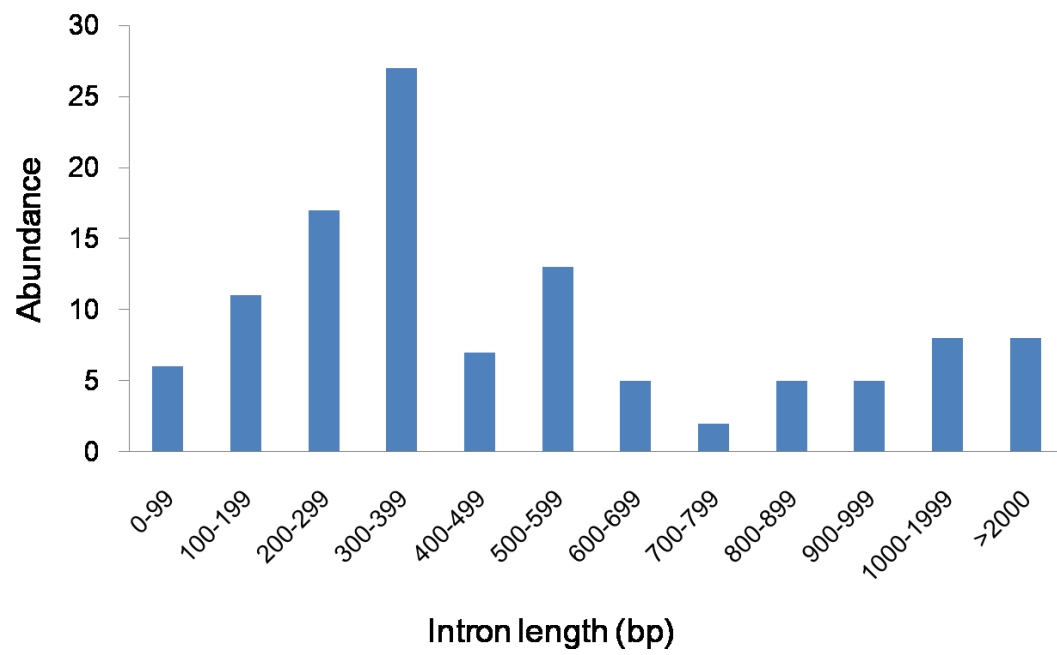

Figure S1 Numerical distribution of intron length in the PxGSTs

Supplement: Additional file 5: Figure S1. — Numerical distribution of intron length in the PxGSTs. [file 12864_2015_1343_MOESM5_ESM.pdf]
