# Supplementary material for: Characterization and expression profiling of glutathione S-transferases in the diamondback moth, Plutella xylostella (L.)
Source: BMC Genomics. 2015 Mar 5;16(1):152. doi: 10.1186/s12864-015-1343-5 (PMC4358871; doi:10.1186/s12864-015-1343-5)
Supplement: Additional file 6: Table S2. — GSH and substrate binding sites of the PxGSTs. [file 12864_2015_1343_MOESM6_ESM.pdf]

Table S2 GSH and substrate binding sites of the PxGSTs

Gene	GSH binding site								Substrate binding site									
PxGSTd1	S14	H55	T56	I57	E69	S70			L106	V110	S111	L114	N115	S118	H122	V166	N169	L210
PxGSTd2	N/A								I102	Y106	Q107	G110	D111	Y114	F118	S163	S166	F205
PxGSTd3	S11	H52	T53	I54	E66	S67			L103	Y107	P108	A111	D112	Y115	F119	T164	T167	F206
PxGSTd4	S12	H53	T54	I55	E67	S68			L104	Y108	D109	A112	V113	Y116	F120	T165	T168	
PxGSTd5	S12	H53	T54	I55	E67	S68			L104	Y108	A109	A112	V113	Y116	F120	T165	T168	F208
PxGSTe1	N/A								S119	F123	T180	T183						
PxGSTe2	N/A								T106	F110	I111	S114	S115	K118	F122	T169	L172	F210
PxGSTe3	N/A								C104	F108	P109	R112	A113	A116	L120	T166	G169	L208
PxGSTe4	S11	H53	T54	V55	D67	S68			T104	F108	P109	R112	G113	E116	F120	T165	S168	
PxGSTe5	S11	H53	T54	I55	D67	S68			S104	F108	P109	R112	A113	A116	F120	T165	S168	F207
PxGSTo1	N/A								S116	Q119	S120	F174	R177					
PxGSTo2	N/A								T123	Q126	G127	W181	R184					
PxGSTo3	N/A								N122	I125	K126	W177	R180					
PxGSTo4	N/A								T123	Q126	N127	W181	Q184					
PxGSTo5	N/A								N116	I119	T120	F174	R177					
PxGSTs1	Y7	I13	Q51	L52	P53	Q64	S65		Y97	W100	F101	I105	P106	I160	A163			
PxGSTs2	Y8	L14	Q50	M51	P52	Q63	S64		N96	R99	A100	A103	Q104	V159	Y162			
PxGSTt1	N/A								H96	R100	A101	E160	Q163					
PxGSTu1	N/A								L100	Y104	A105	S108	A109	L112	F116	S163	T166	L205
PxGSTu2	N/A								S104	Y108	V109	R112	A113	F116	F120	S166	S169	F206
PxGSTz1	S12	C14	R17	Q43	Q56	V57	E69	S70	Q109	Q112	L114	N170	R173					
PxGSTz2	S13	C15	R18	H43	K56	V57	E69	S70	V105	Q109	P110	Q168	N171					
