# Supplementary material for: Characterization and expression profiling of glutathione S-transferases in the diamondback moth, Plutella xylostella (L.)
Source: BMC Genomics. 2015 Mar 5;16(1):152. doi: 10.1186/s12864-015-1343-5 (PMC4358871; doi:10.1186/s12864-015-1343-5)
Supplement: Additional file 9: Table S5. — Primer sequences of qRT-PCR. [file 12864_2015_1343_MOESM9_ESM.pdf]

Table S5 Primer sequences of qRT-PCR

Gene ID[a]	Gene Name	Forward primer (5'-3')	Reverse primer (5'-3')	Product Length(bp)
Px010343	*PxGSTd1*	TATTTGAACCTTTACAGCCCCG	AACACGACCACGATGGAGATG	164
Px015896	*PxGSTd2*	GGAGAACAGATGAAGCCAGAGTA	GTTGACGAGGTATGTGAGGATG	117
Px015897	*PxGSTd3*	GTTTGCGGACTACTTCTACCCCCA	GCCAGCGGCATACTTCTCATCTC	127
Px006286	*PxGSTd4*	CCATAGACCTGTACGAGATGCCCA	CTCTCCAGCCATCAGATCCACAAG	113
Px015631	*PxGSTd5*	GCCATAGAGCTATACGAGATGCAGG	ATCAGGTCGAGGTGGTGGACAG	104
Px014816	*PxGSTe1*	CCAGCCCTCAAAGTCGGTG	CCTGATGATGGGGTCGTCG	111
Px010078	*PxGSTe2*	GGACTACAAGGAGCCCGACC	TTCAGCAAGAACGAAGTTACCATC	115
Px011036	*PxGSTe3*	ATTTTTAGGCAAACTGAGCCAACC	GCAATCCATCTAAGCCAGAAACG	168
Px006106	*PxGSTe4*	TTTTTGGACCACTCATACCTTGCC	CGTTCATGGAGCTGACAGTAGCG	82
Px006105	*PxGSTe5*	CACCGAAGAGTATGTGAAGATGAACC	TGGCGTGGCTGTCCCAAATA	86
Px016897	*PxGSTo1*	TACGAGCAAGGCAAGGGCATC	GGCAGCACTAAAGTTCTCGACAATG	141
Px016898	*PxGSTo2*	CAAGGTTCTTCGGAGGGGAC	CCTTCTGATACGGCAGCACC	84
Px007118	*PxGSTo3*	CTGCCTGAATGGTTCCGAGAGA	TACTTGTCTCCTTGGTCCGTTGG	80
Px015266	*PxGSTo4*	TTCCCAAAACTTTCACAGAGGATACA	TCCACGAATCCTGGCTCCG	121
Px013473	*PxGSTo5*	ACTTCACAGCAGCCTACAACCCT	GAGTGTAGTCTACCATGCCCACTTCT	147
Px009113	*PxGSTs1*	ATTCATCCTGGTCGGCATCATC	TTCTGGACCGCAGCCTTCAC	93
Px009257	*PxGSTs2*	CCAGGTCCACTACGAAGAAGATGAG	GCCGTTGTTCCTCTGCACTATGT	118
Px000759	*PxGSTt1*	ACTGCCAACTACCCTGTCATACG	GTGGGTTTCTTCATAGGGATTCTC	128
Px000790	*PxGSTu1*	TGACTTGTGGAAGATTTCTGAAGATGC	GCCTAATTGGGTGGAAGGGATG	104
Px010993	*PxGSTu2*	GAGCCATTTGTTTCCCGATTCTA	CCACCCATTTGCTACCAGTAAGG	120
Px003659	*PxGSTz1*	ACCACTGATGCCTCAAGACTGCTAC	CGTAGATTAGGACGACCAGGTTTTG	107
Px001225	*PxGSTz2*	ATGGCTGATTTGTGCTTCGTG	GAAAGATGTTTGACTGTCCTGGG	155
